# *Trichomonas vaginalis* infection impairs anion secretion in vaginal epithelium

**DOI:** 10.1371/journal.pntd.0009319

**Published:** 2021-04-16

**Authors:** Jian-Bang Xu, Shen-Jiao Lu, Li-Jiao Ke, Zhuo-Er Qiu, Lei Chen, Hao-Li Zhang, Xi-Yuan Wang, Xiao-Fan Wei, Shuming He, Yun-Xin Zhu, Zhao-Rong Lun, Wen-Liang Zhou, Yi-Lin Zhang

**Affiliations:** 1 School of Life Sciences, Sun Yat-sen University, Guangzhou, China; 2 State Key Laboratory of Respiratory Disease, National Clinical Research Center for Respiratory Disease, Guangzhou Institute of Respiratory Disease, First Affiliated Hospital of Guangzhou Medical University, Guangzhou Medical University, Guangzhou, China; 3 Affiliated Xiaolan Hospital, Southern Medical University, Zhongshan, China; Liverpool School of Tropical Medicine, UNITED KINGDOM

## Abstract

*Trichomonas vaginalis* is a common protozoan parasite, which causes trichomoniasis associated with severe adverse reproductive outcomes. However, the underlying pathogenesis has not been fully understood. As the first line of defense against invading pathogens, the vaginal epithelial cells are highly responsive to environmental stimuli and contribute to the formation of the optimal luminal fluid microenvironment. The cystic fibrosis transmembrane conductance regulator (CFTR), an anion channel widely distributed at the apical membrane of epithelial cells, plays a crucial role in mediating the secretion of Cl^−^ and HCO_3_^−^. In this study, we investigated the effect of *T*. *vaginalis* on vaginal epithelial ion transport elicited by prostaglandin E_2_ (PGE_2_), a major prostaglandin in the semen. Luminal administration of PGE_2_ triggered a remarkable and sustained increase of short-circuit current (*I*_SC_) in rat vaginal epithelium, which was mainly due to Cl^−^ and HCO_3_^−^ secretion mediated by the cAMP-activated CFTR. However, *T*. *vaginalis* infection significantly abrogated the *I*_SC_ response evoked by PGE_2_, indicating impaired transepithelial anion transport via CFTR. Using a primary cell culture system of rat vaginal epithelium and a human vaginal epithelial cell line, we demonstrated that the expression of CFTR was significantly down-regulated after *T*. *vaginalis* infection. In addition, defective Cl^−^ transport function of CFTR was observed in *T*. *vaginalis*-infected cells by measuring intracellular Cl^−^ signals. Conclusively, *T*. *vaginalis* restrained exogenous PGE_2_-induced anion secretion through down-regulation of CFTR in vaginal epithelium. These results provide novel insights into the intervention of reproductive complications associated with *T*. *vaginalis* infection such as infertility and disequilibrium in vaginal fluid microenvironment.

## Author summary

*Trichomonas vaginalis* is a common sexually transmitted parasite that colonized the urogenital mucosa and causes trichomoniasis, a neglected sexually transmitted infection associated with multiple adverse reproductive outcomes in humans. However, the underlying mechanisms remain largely unknown. The epithelial cystic fibrosis transmembrane conductance regulator (CFTR) is an anion channel conducting both Cl^−^ and HCO_3_^−^, which participates in the regulation of luminal fluid microenvironment conducive to the success of reproductive events. Prostaglandin E_2_ (PGE_2_), a bioactive molecule abundant in human seminal fluid, has been demonstrated to exhibit a robust pro-secretory action by activating CFTR in the female genital tract epithelial cells such as endometrial epithelium. These discoveries motivated the authors to investigate the effect of *T*. *vaginalis* infection on exogenous PGE_2_-induced transepithelial transport of electrolytes in vagina. Here, we found that in rat vaginal epithelium, luminal administration of PGE_2_ elicited a response of Cl^−^ and HCO_3_^−^ secretion mediated by cAMP-activated CFTR. However, *T*. *vaginalis* infection impaired transepithelial anion transport evoked by PGE_2_, which is probably related to the defective expression and function of CFTR. These outcomes may complement and expand our knowledge of the complex interaction between *T*. *vaginalis* and the infected host, providing a novel therapeutic strategy for disequilibrium in vaginal fluid microenvironment and infertility induced by *T*. *vaginalis* infection.

## Introduction

The vaginal mucosa is covered with protective stratified squamous epithelial cells served as the sentinels of vaginal defense against potential invading offenders [1]. In the female genital tract, epithelial cells also play vital roles in regulating luminal fluid microenvironments, such as pH, osmolarity and ionic milieu, which is conducive to the success of reproductive events [2]. Vaginal epithelium actively mediates electrolyte transport via multiple ion channels and transporters [2]. Among these ionic-transport proteins, the celebrated cAMP-dependent cystic fibrosis transmembrane conductance regulator (CFTR) is the major anion channel distributed widely in the apical membrane of epithelial cells [3]. The CFTR channel mediates transepithelial Cl^−^ and HCO_3_^−^ transport in both female and male genital tracts including the vagina, playing crucial roles in various reproductive events such as the maintenance of luminal fluid microenvironment homeostasis [4–9]. Conversely, the absence or dysfunction of CFTR results in alterations in the reproductive tract luminal fluid microenvironment and a higher incidence of infertility [10]. The expression and activity of CFTR are dynamically regulated by hormones, neurotransmitters and bioactive signaling factors in the genital tract microenvironment. As one of the predominant prostaglandins in the semen of fertile men, prostaglandin E_2_ (PGE_2_) functions as a critical regulator in diverse reproductive events, such as sperm maturation, ovulation, fertilization, embryo development and early implantation [11–14]. In endometrial epithelium, PGE_2_ has been shown to stimulate CFTR-dependent anion secretory activity [15–17]. However, little is known about the role of the seminal PGE_2_ in mediating electrolyte transport across the vaginal epithelium.

*Trichomonas vaginalis* is a flagellated parasite commonly colonizing the vagina and causes trichomoniasis, one of the most prevalent sexually transmitted infections in humans [18,19]. *T*. *vaginalis* usually harbors *T*. *vaginalis* virus and *Mycoplasma hominis*, which disrupts the equilibrium of lactobacilli-dominant vaginal microbiota and synergistically augments the pro-inflammatory responses in both reproductive tracts including the vagina and prostate [20–23]. Trichomoniasis has been demonstrated to be associated with severe adverse reproductive outcomes, such as infertility and multiple pregnancy complications [24–26], whilst the underlying mechanisms remain largely unclear. Recent studies showed that infection with pathogens including *Campylobacter jejuni* [27] and *Toxoplasma gondii* [28] impaired the function of epithelial Cl^−^ secretion mediated by CFTR, suggesting a close correlation between pathogen infection and aberrant function of CFTR in host epithelial cells. As the prerequisite to colonization, *T*. *vaginalis* adheres to the host vaginal epithelial cells and initiates the inflammatory response [18,23,29,30]. Our previous work has revealed that in human vaginal epithelial cells, *T*. *vaginalis* infection elicited the down-regulation of CFTR in a cysteine protease-dependent manner, thereby mediating epithelial inflammation via intracellular Cl^−^ signaling pathways [30]. These observations indicated the putative impairment of ion transport pathways and the imbalanced vaginal luminal fluid microenvironment after *T*. *vaginalis* infection. Therefore, this study aims to investigate the effect of *T*. *vaginalis* infection on the CFTR-dependent anion secretion of vaginal epithelium induced by exogenous PGE_2_ and elucidate the underlying mechanisms.

## Materials and methods

### Ethics statement

Animal care and experimentation were performed following the guidelines described by the Sun Yat-sen University Animal Use Committee (Guangzhou, China). All procedures were approved by the Sun Yat-sen University Animal Use Committee (Guangzhou, China).

### Parasites and animals

*T*. *vaginalis* strain CPO 02 was obtained as a kind gift from Prof. Zhao-Rong Lun (School of Life Sciences, Sun Yat-sen University, Guangzhou, China), and was cultured as previously described [30]. Briefly, the *T*. *vaginalis* trophozoites were cultured in Diamond’s Trypticase Yeast Extract Maltose (TYM) medium (Huankai Microbial, China), supplemented with 10% (vol/vol) heat-inactivated fetal bovine serum (FBS, Tianhang Biotechnology, China), penicillin/streptomycin (100 U/mL/100 μg/mL, Hyclone, USA) at 37°C in an atmosphere of 5% CO_2_ [31]. Only the parasites in a logarithmic phase of growth were used for assays in this study.

Female Sprague-Dawley (SD) rats, weighing 200–250 g, were purchased from the Laboratory Animal Center of Sun Yat-sen University (Guangzhou, China). The rats received a subcutaneous injection of 0.5 mg β-estradiol (Sigma Aldrich, USA) every second day to initiate a persistent estrus. The rats were subsequently infected intravaginally with 3×10^7^ *T*. *vaginalis* trophozoites once a day in the next consecutive two days, whereas the control group rats were pipetted intravaginally with an equal volume of phosphate-buffered saline (PBS, pH 7.4). The rats were euthanized 24 h following the last infection, and the excised vaginal mucosa was used for the measurement of short-circuit current (*I*_SC_).

### Primary culture of rat vaginal epithelial cells

Female SD rats were euthanatized by CO_2_ asphyxia and the freshly excised vaginal tissues were washed and cut into small pieces under antiseptic conditions. The finely minced tissues were subsequently digested enzymatically twice (1 h for each time) with type I collagenase (0.5 mg/mL, C0130, Sigma-Aldrich, USA) dissolved in DMEM/F12 (Gibco, USA) at 37°C, with gentle agitation. The digested tissues were further dissociated and dispersed by repeatedly pipetting and centrifuged at 500 *g* for 30 s. Afterward, the isolated cell clusters were resuspended and washed twice with DMEM/F12 (Gibco, USA) medium containing 10% FBS (Gibco, USA). The fragments were collected and placed in the six-well plate, then humidified in keratinocyte serum-free medium (K-SFM, Gibco, USA) supplemented with bovine pituitary extract (50 μg/mL, Gibco, USA), recombinant epidermal growth factor (5 ng/mL, Gibco, USA), 100× insulin-transferrin-selenium solution (1×, Gibco, USA), cholera toxin (Sigma Aldrich, USA) and penicillin/streptomycin (100 U/mL/100 μg/mL, Hyclone, USA), in an atmosphere of 5% CO_2_ at 37°C. After 24 h, the fragments were washed and cells were cultured with the supplemented K-SFM medium. On day 5, the cells were used for immunofluorescence staining or incubated with live *T*. *vaginalis* (1×10^6^) for Western blot assay.

### Cell culture

Human vaginal epithelial cell line VK2/E6E7 cells was purchased from Beijing ZhongYuan Ltd (China) and were cultured in K-SFM supplemented with bovine pituitary extract (50 μg/mL, Gibco, USA), recombinant epidermal growth factor (0.1 ng/mL, Gibco, USA), 1% (v/v) penicillin-streptomycin (Hyclone, USA) and 0.4 mM CaCl_2_ (Guangzhou Chemical Pharmaceutical Factory, China) at 37°C with 5% CO_2_ in a humidified atmosphere.

### Measurement of ion transport using the Ussing chamber

To measure the transepithelial *I*_SC_, *in vitro* Ussing chamber experiments equipped with vaginal tissues of female SD rats was carried out as described previously [7,8]. Briefly, the freshly isolated rat vaginal mucosae were mounted vertically between two halves of the Ussing chambers, with an available permeation area of 0.45 cm^2^. Each side of the mucosal sheet was bathed symmetrically with Krebs-Henseleit (K-H) buffer bubbled continuously at 37°C with carbogen (95% O_2_, 5% CO_2_) to maintain a constant pH of 7.4. The normal K-H solution (pH 7.4) was composed as follows (in mM): 117 NaCl, 4.7 KCl, 1.2 MgSO_4_·7H_2_O, 24.8 NaHCO_3_, 1.2 KH_2_PO_4_, 2.5 CaCl_2_, and 11.1 D-glucose (Guangzhou Chemical Pharmaceutical Factory, China). Two pairs of Ag/AgCl electrodes were used to measure the basal transepithelial potential exhibited by different epithelia. The electrodes were coupled to a multichannel voltage-current clamp (MODEL VCC MC6, Physiologic Instruments, USA) and connected to each chamber by the agar-salt bridges made of 3 M KCl and 3% (wt/vol) agar. The *I*_SC_ (in μA) was recorded continuously when the voltage of the tissue was clamped at 0 mV by the automatic voltage clamp amplifier. When the *I*_SC_ was stable, different stimulations were pipetted into the apical side or basolateral side of the epithelium as needed. The changes in *I*_SC_ (Δ*I*_SC_) were recorded and normalized for the opening area of the Ussing chamber (in ΔμA/cm^2^).

In ion substitution experiments, gluconate was used to replace Cl^−^ in Cl^−^-free K-H solution, whereas N-2-hydroxyethylpiperazine-N-2-ethane sulphonic acid (HEPES, Mbchem Technology, China) was used to replace NaHCO_3_ in HCO_3_^−^-free K-H solution, which was gassed with 100% O_2_ throughout the experiment.

### Immunofluorescence staining

The primary cultured rat vaginal epithelial cells or the VK2/E6E7 cells were seeded on sterile glass coverslips. On day 5, the primary cells were used to certify the location of keratin and CFTR. For *T*. *vaginalis* infection, the primary cells were infected with 1 × 10^6^ *T*. *vaginalis* for 3 h and the VK2/E6E7 cells were infected with 2 × 10^5^ *T. vaginalis* for 3 h before the immunofluorescence assay. The samples were washed with pre-cold PBS and then fixed with 4% paraformaldehyde for 20 min, permeabilized with 0.1% Triton X-100 in PBS for 5 min, and blocked with 3% bovine serum albumin (BSA) for 1 h at room temperature. The cells were then incubated at 4°C overnight with mouse anti-Pan-Keratin (#4545, Cell Signalling Technology, USA) or mouse anti-CFTR antibody (ab2784, Abcam, UK), followed by Alexa Fluor 488-labeled donkey anti-mouse IgG (A-21202, Thermo Fisher Scientific, USA) for 1 h at room temperature. The nuclei were stained with 4’,6-diamidino-2-phenylindole (DAPI, C1006, Beyotime, China). The fluorescence was examined by laser scanning confocal microscopy (TCS-SP5, Leica, Germany).

### Western blot

Western blot was performed as described previously [30]. The primary antibody against CFTR (1:500, ab2784) was purchased from Abcam (UK), and the antibody against β-actin (1:1000, #4970) was purchased from Cell Signalling Technology (USA).

### Intracellular Cl^−^ measurement

The measurement of intracellular Cl^−^ was performed as previously described [30]. Briefly, the primary cultured rat vaginal epithelial cells or the VK2/E6E7 cells were grown on glass cover slips with or without *T*. *vaginalis* infection for 3 h. The changes of fluorescence intensity of the Cl^−^ indicator dye, MQAE, was recorded using an imaging system (Olympus, IX83, Tokyo, Japan).

### Statistical analysis

All statistical data were summarized as the mean ± standard deviation (S.D). Student’s two tailed *t* test was used for pairwise comparisons, and one-way ANOVA with Bonferroni was used to test for multiple comparisons. Data were analyzed using Origin Pro 8.0 software (OriginLab, USA), and *P* values < 0.05 were considered significantly different. The numerical data used in all figures are included in S1 Data.

## Results

### Characteristics of *I*_SC_ response elicited by luminal administration of PGE_2_ in rat vaginal epithelium

**Fig 1:**
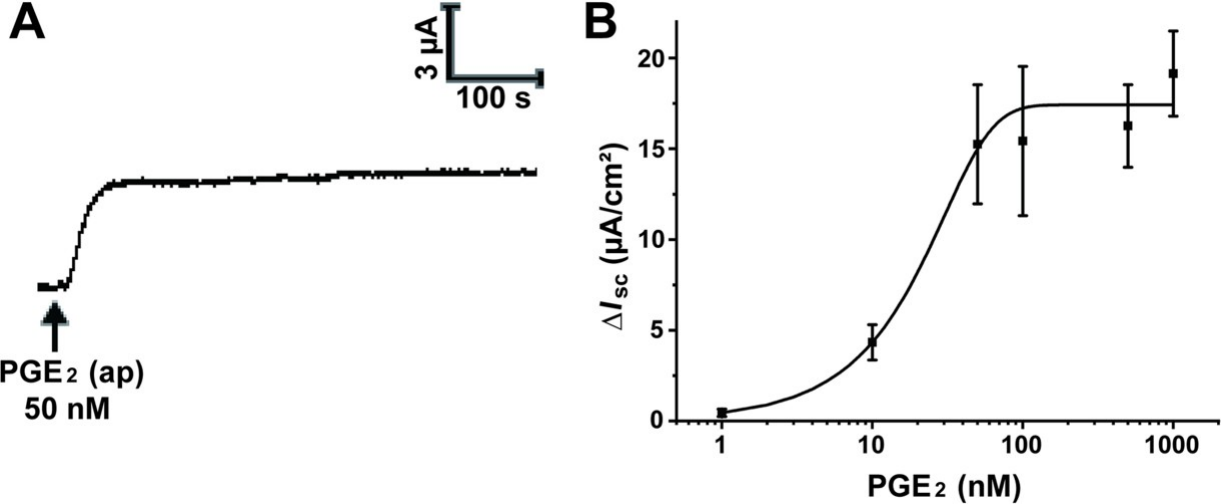
Apical administration of prostaglandin E_2_ (PGE_2_) stimulated an increase in short-circuit current (*I*_SC_) across rat vaginal epithelium. (A) Representative trace showing the effect of the apical (ap) application of PGE_2_ (50 nM) on *I*_SC_ response in rat vaginal epithelium. (B) The concentration-dependent curve of PGE_2_ induced *I*_SC_ responses with a half-maximal effective concentration (EC50) of 24.9 nM. Symbols and bars indicate the means ± S.D. (*n* = 3–5).

To explore the potential effect of seminal PGE_2_ on transepithelial electrolyte transport in the vagina, we measured the *I*_SC_ using the Ussing chamber system. Apical administration of PGE_2_ (50 nM) induced a remarkable *I*_SC_ response characterized by a rapid increase and a long duration (Fig 1A). In addition, luminal PGE_2_-induced *I*_SC_ response was in a concentration-dependent manner with a half-maximal effective concentration (EC50) of 24.9 nM (Fig 1B). Thus, a concentration of 50 nM was chosen for the subsequent experiments.

An increase in *I*_SC_ response represents net anion secretion or net cation absorption. Our previous study has shown that amiloride, a potent inhibitor of the epithelial Na^+^ channel (ENaC), significantly decreased the basal *I*_SC_ of rat vaginal epithelial cells [8], indicating the important role of ENaC-mediated Na^+^ influx in vaginal epithelial ion transport. Therefore, we initially investigated whether seminal PGE_2_ promoted the absorption of Na^+^ and thereby increased the *I*_SC_ response. As is shown in S1 Fig, luminal pretreatment with amiloride (100 μM) failed to affect the *I*_SC_ response induced by PGE_2_, excluding the involvement of ENaC in this process. On the contrary, the PGE_2_-elicited *I*_SC_ responses were markedly suppressed in Cl^−^-free, HCO_3_^−^-free or both Cl^−^ and HCO_3_^−^-free solution (S2 Fig), revealing that luminal administration of PGE_2_ in vaginal epithelium stimulated transepithelial secretion of Cl^−^ and HCO_3_^−^.

The CFTR channel reportedly mediates the transepithelial anion transport across the apical membrane of epithelium, including the vaginal epithelium [7,8]. We next sought to validate the involvement of CFTR in the *I*_SC_ response induced by the luminal administration of PGE_2_. Both the non-selective Cl^−^ channel blocker DPC (1 mM) and the selective CFTR blocker CFTR_inh-172_ (10 μM) remarkably inhibited the luminal PGE_2_-elicited increase of *I*_SC_ (S3 Fig), suggesting that the *I*_SC_ response was mediated by activation of CFTR. Considering that CFTR is activated by elevation of intracellular cAMP, we further investigated the cellular mechanism underlying seminal PGE_2_-induced CFTR activation. Pretreatment with either forskolin (20 μM), an activator of adenylate cyclase, or MDL-12330A (10 μM), an inhibitor of adenylate cyclase, potently abolished the *I*_SC_ response (S3 Fig), which implied that luminal PGE_2_ facilitated anion secretion by activating CFTR via adenylate cyclase-cAMP signaling pathway.

Finally, we also verified the involvement of Na^+^-K^+^-2Cl^−^ cotransporter (NKCC), which mediates Cl^−^ uptake across the basolateral membrane and accumulate Cl^−^ intracellularly [32,33]. Notably, the apical PGE_2_-stimulated *I*_SC_ response was significantly diminished by bumetanide (100 μM), an inhibitor of NKCC (S4 Fig), confirming the critical role of NKCC in luminal PGE_2_-induced *I*_SC_ response by supplying Cl^−^.

### *T*. *vaginalis* infection attenuated anion secretion mediated by luminal PGE_2_ in rat vaginal epithelium

**Fig 2:**
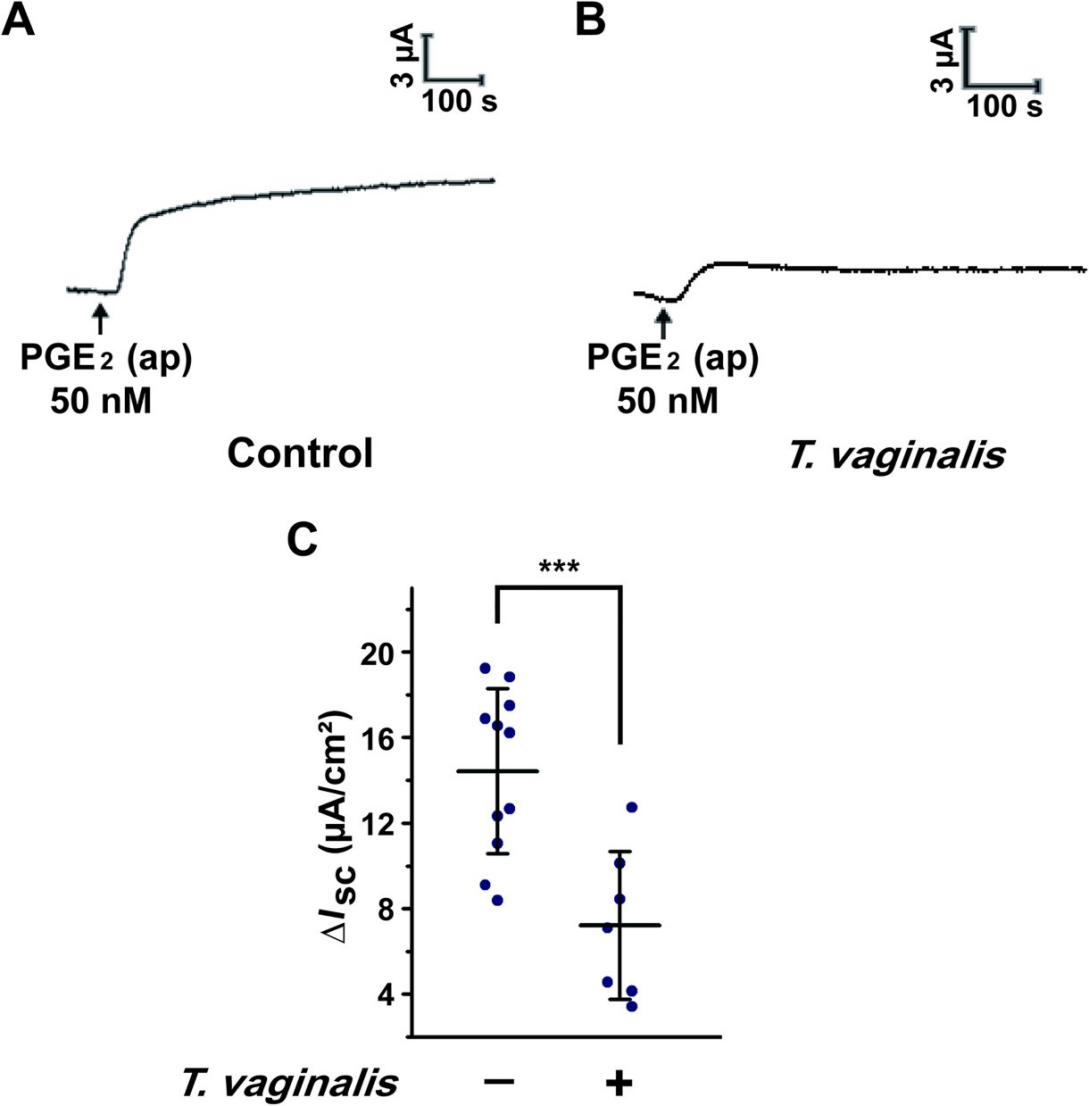
*Trichomonas vaginalis* infection impaired the anion transport induced by prostaglandin E_2_ (PGE_2_) in rat vaginal epithelium. (A-B) Representative trace showing the short-circuit current (*I*_SC_) response induced by apical (ap) PGE_2_ (50 nM) with (B) or without (A) intravaginal *T*. *vaginalis* infection in rats. (C) Statistical analysis showing the effect of *T*. *vaginalis* infection on the *I*_SC_ currents induced by apical PGE_2_ (50 nM) in rat vaginal epithelium. Symbols and bars indicate the mean ± S.D. (*n* = 7–11, *** *P* < 0.001).

*T*. *vaginalis* infection is associated with an abnormal vaginal luminal fluid microenvironment. To investigate the effect of *T*. *vaginalis* infection on the vaginal transepithelial anion secretion induced by luminal PGE_2_, we established a rat model of *T*. *vaginalis* infection. As illustrated in Fig 2, compared with the control group, *T*. *vaginalis* infection remarkably attenuated the *I*_SC_ response elicited by apical administration of PGE_2_, revealing that *T*. *vaginalis* infection impaired transepithelial anion secretion in rat vaginal epithelium.

### *T*. *vaginalis* infection triggered down-regulation of CFTR in vaginal epithelium

**Fig 3:**
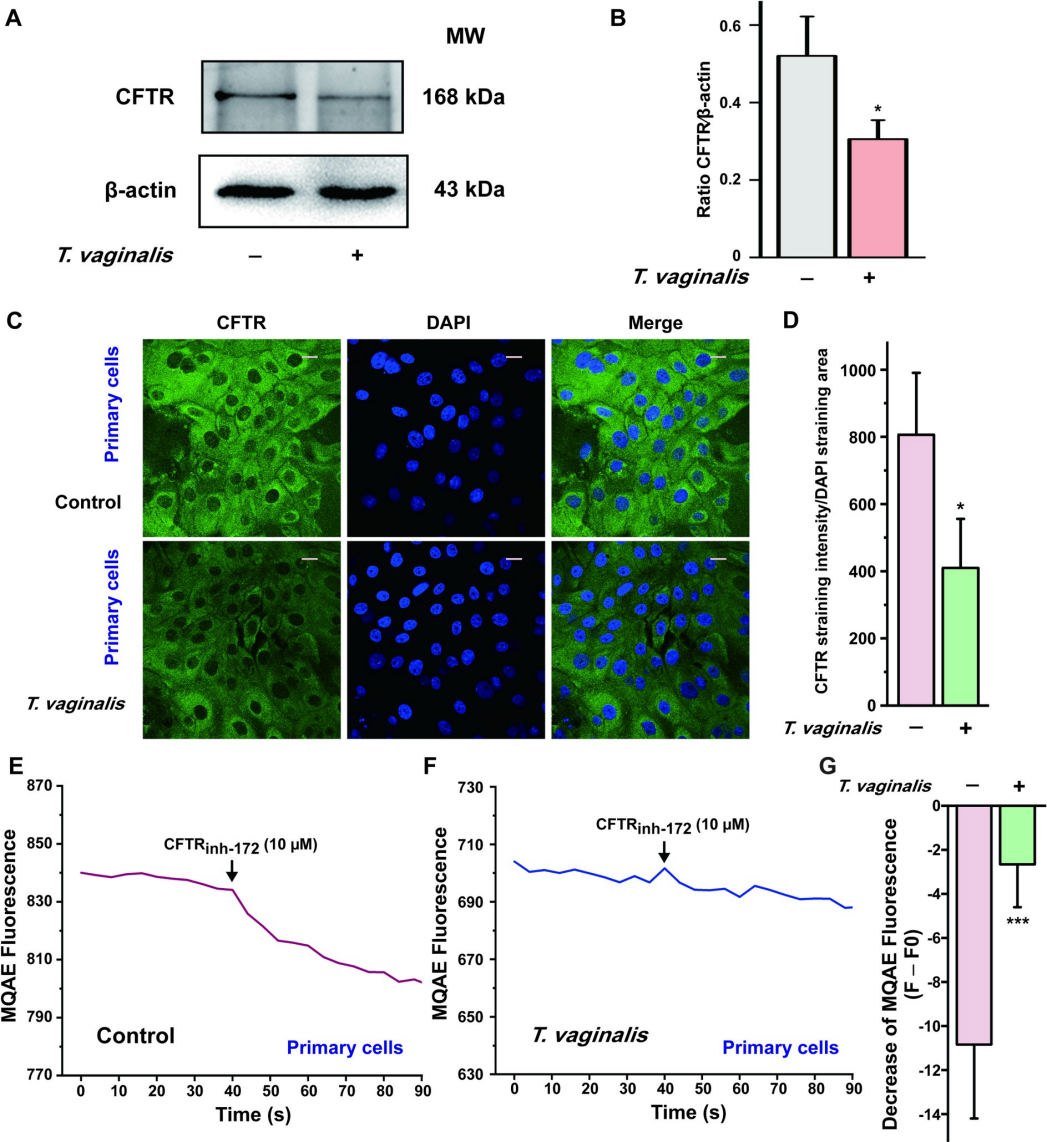
*Trichomonas vaginalis* infection induced down-regulation of cystic fibrosis transmembrane conductance regulator (CFTR) in the primary cultured rat vaginal epithelial cells. (A) Representative blots showing the expression of CFTR in primary cultured vaginal epithelial cells infected with live 1 × 10^6^ *T*. *vaginalis* for 3 h, using β-actin as a loading control. MW, molecular weight. (B) Statistical analysis of Western blot (CFTR/β-actin ratio) showing the effect of *T*. *vaginalis* infection on the expression of CFTR. Symbols and bars indicate the mean ± S.D. (*n* = 3, * *P* < 0.05 versus the non-infected group). (C) Immunofluorescence images showing the expression of CFTR in primary cultured rat vaginal epithelial cells, in the absence or presence of 1 × 10^6^ *T*. *vaginalis* infection, with (D) the corresponding quantification analysis (*n* = 3, * *P* < 0.05 versus the non-infected group). Scale bar = 20 μm. (E) Representative trace showing the change of MQAE fluorescence elicited by CFTR_inh-172_ (10 μM) in primary cultured rat vaginal epithelial cells. (F) Representative trace showing the change of MQAE fluorescence elicited by CFTR_inh-172_ (10 μM) after 1 × 10^6^ *T*. *vaginalis* infection for 3 h. (G) Statistical analysis showing the change of MQAE fluorescence intensity elicited by CFTR_inh-172_ (10 μM), with or without *T*. *vaginalis* infection. Symbols and bars indicate the mean ± S.D. (*n* = 31–42 cells for each group, *** *P* < 0.001 versus the non-infected group).

**Fig 4:**
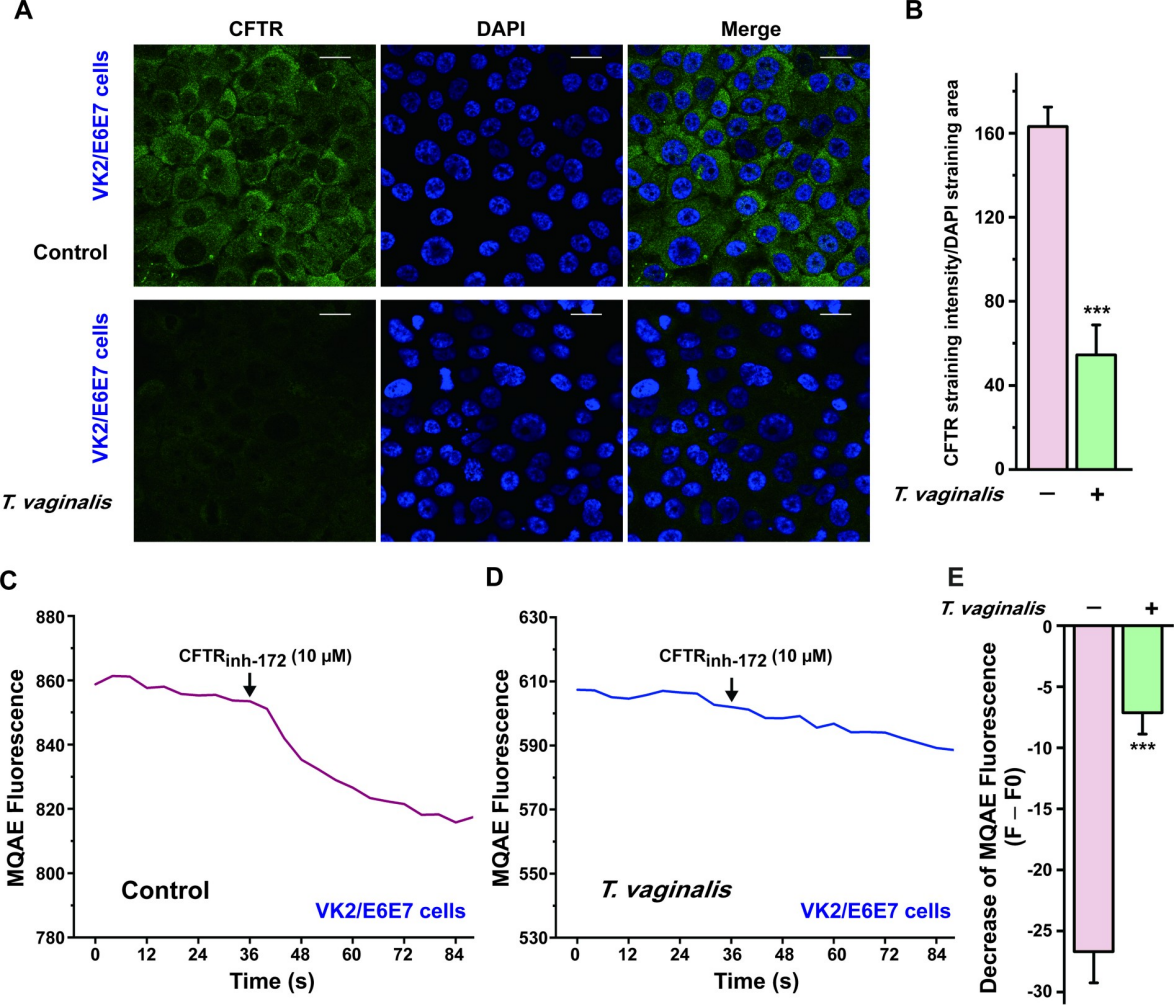
*Trichomonas vaginalis* infection induced down-regulation of cystic fibrosis transmembrane conductance regulator (CFTR) in human vaginal epithelial VK2/E6E7 cells. (A) Immunofluorescence images showing the expression of CFTR in VK2/E6E7 cells, in the absence or presence of 2×10^5^ *T*. *vaginalis* infection for 3 h, with (B) the corresponding quantification analysis (*n* = 3, *** *P* < 0.001 versus the non-infected group). Scale bar = 20 μm. (C) Representative trace showing the change of MQAE fluorescence elicited by CFTR_inh-172_ (10 μM) in VK2/E6E7 cells. (D) Representative trace showing the change of MQAE fluorescence elicited by CFTR_inh-172_ (10 μM) after 2×10^5^ *T*. *vaginalis* infection for 3 h. (E) Statistical analysis showing the change of MQAE fluorescence intensity elicited by CFTR_inh-172_ (10 μM), with or without *T*. *vaginalis* infection. Symbols and bars indicate the mean ± S.D. (*n* = 39–41 cells for each group, *** *P* < 0.001, versus the non-infected group).

In the light of the crucial role of CFTR in mediating anion secretion induced by luminal administration of PGE_2_ in vaginal epithelium, we then tested the effect of *T*. *vaginalis* infection on the expression and function of CFTR. We successfully established a primary rat vaginal epithelial cell culture system. As shown in S5 Fig, the primary cultured vaginal epithelial cells retained the epithelial morphology in light microscopy, with positive expression of CFTR and pan-keratin, a marker for epithelial cells. After *T*. *vaginalis* infection, however, the expression of CFTR was significantly down-regulated in primary cultured vaginal epithelial cells by using Western blot and immunofluorescence staining analysis (Fig 3A–3D). Furthermore, we also evaluate the effect of *T*. *vaginalis* infection on CFTR function using the intracellular Cl^−^ measurement technique. Treatment with CFTR_inh-172_ (10 μM) elicited a decrease of MQAE fluorescence in the primary cultured rat vaginal epithelial cells, which represented an increase of intracellular Cl^−^ concentration owing to the dysfunction of CFTR as described previously [30]. Nevertheless, the decrease of MQAE fluorescence elicited by CFTR_inh-172_ was significantly restrained after *T*. *vaginalis* infection (Fig 3E–3G), indicating that *T*. *vaginalis* impaired the Cl^−^ transport function of CFTR in rat vaginal epithelial cells. Similar results were observed in the human vaginal epithelial VK2/E6E7 cells (Fig 4). Taken together, these results demonstrated that *T*. *vaginalis* infection triggered defective CFTR expression and function in vaginal epithelium, which might be the probable cause of the decreased PGE_2_-elicited *I*_SC_ response after *T*. *vaginalis* infection.

## Discussion

**Fig 5:**
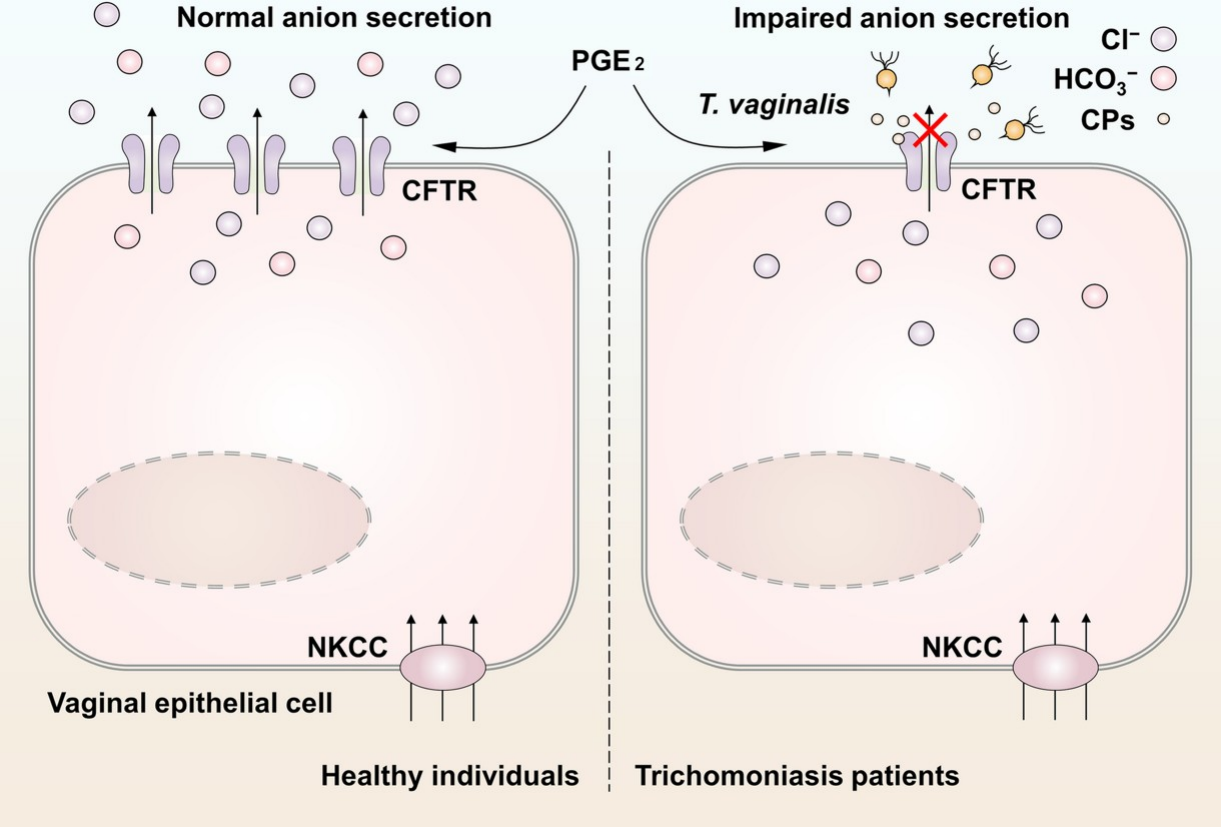
Schematic model of the impaired anion secretion triggered by *Trichomonas vaginalis* infection in vaginal epithelium. Seminal prostaglandin E_2_ (PGE_2_) facilitated anion secretion by activating cystic fibrosis transmembrane conductance regulator (CFTR) in vaginal epithelium. The basolateral Na^+^-K^+^-2Cl^−^ cotransporter (NKCC) guaranteed the maintenance of steady anion secretion by supplying Cl^−^. After *T*. *vaginalis* infection, the CFTR was markedly down-regulated via cysteine proteases secreted by the pathogens, which restrained the exogenous PGE_2_-induced anion secretion. This may be a cause of the abnormal vaginal fluid microenvironment in patients with trichomoniasis.

*T*. *vaginalis* is a common sexually transmitted eukaryotic parasite, which adheres to vaginal epithelial cells and causes trichomoniasis [18,23,29]. Although polarized epithelial cells in the female reproductive tract have been recognized as the sentinels of immune protection [1], the mechanisms underlying the host-parasite interaction are still not clearly understood. In this study, we established a rat model of *T*. *vaginalis* infection and elucidated that *T*. *vaginalis* infection impaired the transepithelial anion secretion triggered by luminal PGE_2_ via down-regulation of CFTR (Fig 5).

PGE_2_ is synthesized from arachidonic acid via the cyclooxygenase pathway in various cell types [34]. In fertile men, a high level of PGE_2_ with a concentration of approximate 70 mg/L was detectable in the semen [11,35]. Seminal prostaglandins have been shown to protect sperm from immunological damage in the male genital tract and actively regulate sperm maturation and sperm motility [12,14,36]. A lower level of PGE_2_ was observed in the seminal plasma of infertile men with genital tract infection than that in fertile men, revealing the important role of PGE_2_ in male reproductive health [37]. In the female reproductive system, PGE_2_ also plays a pivotal role in various physiological events, including ovulation, fertilization, embryo development, early implantation, and provides a tolerogenic immune microenvironment [13,14]. Previous research has demonstrated that PGE_2_ induced a potent and sustained increase of *I*_SC_ in the female genital tract epithelial cells such as endometrial epithelium [16,17], suggesting that exogenous PGE_2_ from the ejaculated semen might be implicated in mediating ion transport processes in vagina mucosa after coitus. Notably, our results showed that the luminal administration of PGE_2_ elicited anion (mainly Cl^−^ and HCO_3_^−^) secretion across the rat vaginal epithelium. This response was presumably via activation of the cAMP-dependent CFTR channel, which is consistent with previous observations in human bronchial epithelial cells [38]. Moreover, basolateral NKCC was also involved in PGE_2_-elicited anion transport, which supported the apical Cl^−^ secretion via cellular supply of Cl^−^ [5,32,39,40]. Transepithelial anion secretion is responsible for contributing to the formation of the optimal luminal fluid microenvironment in the vagina. On one hand, Cl^−^ secretion provides the osmotic driving force for passive H_2_O transport [39], which leads to vaginal lubrication [7,8]. On the other hand, HCO_3_^−^ secretion to the vaginal lumen might be conducive to regulating luminal pH homeostasis and sperm functions [41,42]. An optimal luminal vaginal pH is essential for fertilization since sperm are susceptible to the acidic pH after deposition [43]. Thus, our results indicated that during semen deposition in the vagina, the seminal PGE_2_ promoted anion secretion across the vaginal epithelial cells, which might be indispensable for fluid secretion and regulation of luminal pH and sperm motility.

Recent studies have demonstrated the impairment of transepithelial Cl^−^ transport after infection with pathogens including *C*. *jejuni* [27], *T*. *gondii* [28], and influenza virus [44]. Here in our study, we verified that *T*. *vaginalis* infection significantly inhibited anion secretion elicited by the luminal administration of PGE_2_ in rat vaginal epithelium. As the major channel mediating anion transport, the apically located CFTR was reportedly down-regulated after pathogenic infection in host epithelial cells [27,44,45]. Consistent with these observations, we showed that *T*. *vaginalis* infection triggered down-regulation of CFTR in primary cultured rat vaginal epithelial cells, which may probably be the cause of impaired anion secretion. Our previous work has revealed that the cysteine proteases secreted by *T*. *vaginalis* degraded CFTR protein in human vaginal epithelial VK2/E6E7 cells [30]. We speculated that *T*. *vaginalis* infection-induced impairment of transepithelial anion secretion may also be attributed to the effects of cysteine proteases secreted by the parasites. Previous studies have highlighted the linkage between defect of CFTR and impaired host defense function of epithelial cells and neutrophils [46,47]. Here, our findings extended the previously recognized involvement of cysteine proteases in *T*. *vaginalis* immune-evasive behaviors, which was not solely associated with the degradation of antibodies [23], but also mediated host-defense failure via degradation of CFTR expressed in vaginal epithelial cells and neutrophils.

In addition to inflammation, multiple complications including infertility, are associated with trichomoniasis. Clinical data showed that the prevalence of *T*. *vaginalis* infection in infertile women was significantly higher than that in the control group [48]. Additionally, trichomoniasis-related fertility disorders may be ascribed to the phagocytosis of sperm cells during their journey along the female reproductive tract [25,49]. Previous investigations showed that a PGE_2_-rich microenvironment protects sperm from phagocytosis [50]. Our findings indicated that the optimal luminal fluid microenvironment modulated by PGE_2_ could be destroyed by *T*. *vaginalis* infection. This might have, at least partially, accounted for the protective role of PGE_2_ and the pathogenesis of trichomoniasis-related sperm phagocytosis. As the sexually transmitted protozoan, *T*. *vaginalis* may be transmitted from both male or female carriers to their partners through sexual intercourse. Considering the evidence of the acute and chronic inflammation in the prostate after *T*. *vaginalis* infection [20,51], we speculated that defective CFTR expression and function may also exist in the upper genital tract epithelium of men infected with *T*. *vaginalis*, although further studies are required. Additionally, sexual transmission of *T*. *vaginalis* complicates the scenarios of *T*. *vaginalis*-PGE_2_-CFTR interactions, leading to different short-term impact and long-term outcomes, especially when co-infected with other bacterial pathogen or in symbiosis with *T*. *vaginalis* virus and *M*. *hominis* [22,23]. These situations synergistically worsen the inflammatory damage to the genital tract. It should be noted that new evidence supports the atypical locations of trichomonads including *T*. *vaginalis* and zoonotic trichomonads in the human respiratory tract [52,53]. In the light of our previous work that CFTR dysfunction elicited chronic airway inflammation via Cl^−^-sensing kinase [54], the presented data as we showed here may have far-reaching implications beyond non-viral sexually transmitted diseases since acquired defects in CFTR might also be implicated in pulmonary trichomoniasis.

In conclusion, this study revealed that *T*. *vaginalis* infection impaired the PGE_2_-elicited anion transport via down-regulation of CFTR in vaginal epithelium, confirming the crucial role of CFTR in the host-parasite interaction. Our results provide valuable insights into a better understanding of the pathogenesis of trichomoniasis and offer a novel therapeutic strategy for *T*. *vaginalis* infection via restoration of epithelial CFTR function.

## Supporting information

S1 Fig Luminal prostaglandin E_2_ (PGE_2_)-induced short-circuit current (*I*_SC_) response was not affected by amiloride. (A) Representative trace showing the *I*_SC_ response induced by apical (ap) PGE_2_ (50 nM) in vaginal epithelium. (B) Representative trace showing the *I*_SC_ response induced by apical (ap) PGE_2_ (50 nM) in the presence of amiloride (100 μM, ap) in rat vaginal epithelium. (C) Statistical analysis showing the effect of apically applied amiloride on the *I*_SC_ response induced by PGE_2_. Symbols and bars indicate the means ± S.D. (*n* = 4, ns = no significant).
(TIF)

S2 Fig Luminal prostaglandin E_2_ (PGE_2_)-induced short-circuit current (*I*_SC_) response was electrogenic anion secretion. (A-D) Representative trace showing the *I*_SC_ response induced by apical (ap) addition of PGE_2_ (50 nM) in normal K-H solution (A), Cl^−^ -free K-H solution (B), HCO_3_^−^- free K-H solution (C), and Cl^−^ and HCO_3_^−^ both free K-H solution (D). (E) Statistical analysis showing the *I*_SC_ response in rat vaginal epithelium induced by PGE_2_ (50 nM) in different K-H solutions. Symbols and bars indicate the mean ± S.D. (*n* = 3, ** *P* < 0.01, *** *P* < 0.001 versus the normal K-H solution group).
(TIF)

S3 Fig Luminal prostaglandin E_2_ (PGE_2_)-induced anion secretion was mediated by cystic fibrosis transmembrane conductance regulator (CFTR). (A-D) Representative trace showing the short-circuit current (*I*_SC_) response induced by apical (ap) addition of PGE_2_ (50 nM) after treatment with the non-selective Cl^−^ channels blocker DPC (1 mM, ap) (A), the selective CFTR blocker CFTR_inh-172_ (10 μM, ap) (B), the adenylate cyclase activator forskolin (FSK, 20 μM, ap) (C), or the adenylate cyclase inhibitor MDL-12330A (MDL, 10 μM, ap) (D). (E) Statistical analysis showing the effect of inhibitors on the *I*_sc_ response induced by PGE_2_. Symbols and bars indicate the mean ± S.D. (*n* = 3–6, * *P* < 0.05, ** *P* < 0.01 versus the PGE_2_ group).
(TIF)

S4 Fig Luminal prostaglandin E_2_ (PGE_2_)-induced short-circuit current (*I*_SC_) response was mediated by Na^+^-K^+^-2Cl^−^ cotransporter (NKCC). (A) Representative trace showing the *I*_SC_ response induced by apical (ap) PGE_2_ (50 nM) in the presence of basolateral (bl) administration of bumetanide (100 μM), an inhibitor of the Na^+^-K^+^-2Cl^−^ cotransporter, in rat vaginal epithelium. (B) Statistical analysis showing the effect of basolateral applied bumetanide on the *I*_SC_ response induced by PGE_2_. Symbols and bars indicate the means ± S.D. (*n* = 4, *** *p* < 0.001 versus the PGE_2_ group).
(TIF)

S5 Fig Characteristic of the primary cultured rat vaginal epithelial cells. (A) Light microscope image of primary cultured rat vaginal epithelial cells (Day 5). Scale bar = 50 μm. (B) Representative blots showing the expression of cystic fibrosis transmembrane conductance regulator (CFTR) in primary cultured vaginal epithelial cells (Day 4). (C) Immunofluorescence images showing the expression of keratin, the marker of epithelial cells, and CFTR in primary cultured rat vaginal epithelial cells (Day 4), with the negative control. Scale bar = 10 μm.
(TIF)

S1 Data Excel spreadsheet containing, in separate sheets, the underlying raw data and statistical analysis for Figs 1B, 2C, 3B, 3D–3G, 4B–4E, S1B, S2E, S3E, S4B. (XLSX)
