# Supplementary figures and images for: Trichomonas vaginalis infection impairs anion secretion in vaginal epithelium
Source: PLoS Negl Trop Dis. 2021 Apr 16;15(4):e0009319. doi: 10.1371/journal.pntd.0009319 (PMC8051796; doi:10.1371/journal.pntd.0009319)

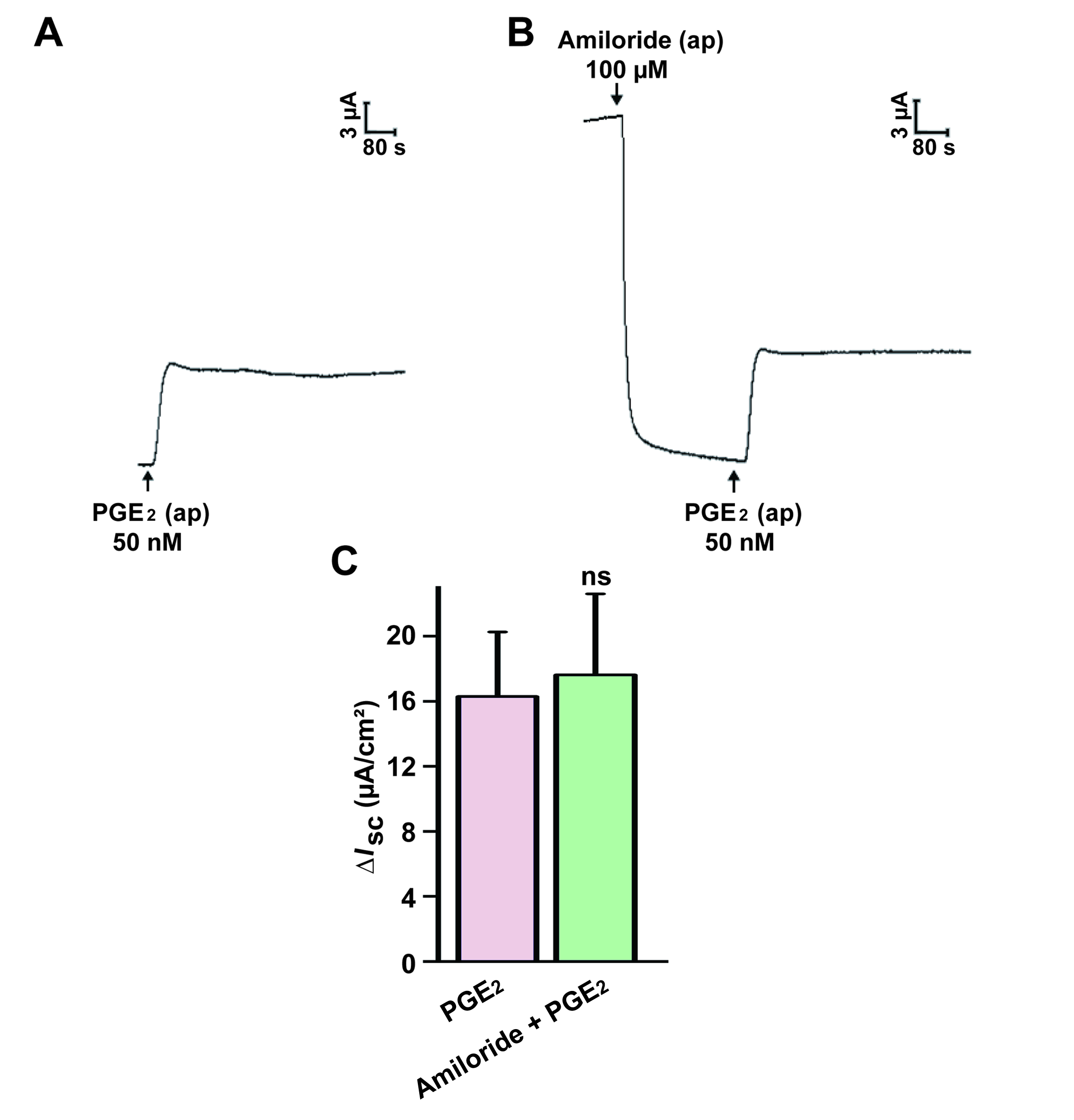

Supplement: S1 Fig — (A) Representative trace showing the ISC response induced by apical (ap) PGE2 (50 nM) in vaginal epithelium. (B) Representative trace showing the ISC response induced by apical (ap) PGE2 (50 nM) in the presence of amiloride (100 μM, ap) in rat vaginal epithelium. (C) Statistical analysis showing the effect of apically applied amiloride on the ISC response induced by PGE2. Symbols and bars indicate the means ± S.D. (n = 4, ns = no significant). (TIF) [file pntd.0009319.s001.tif]

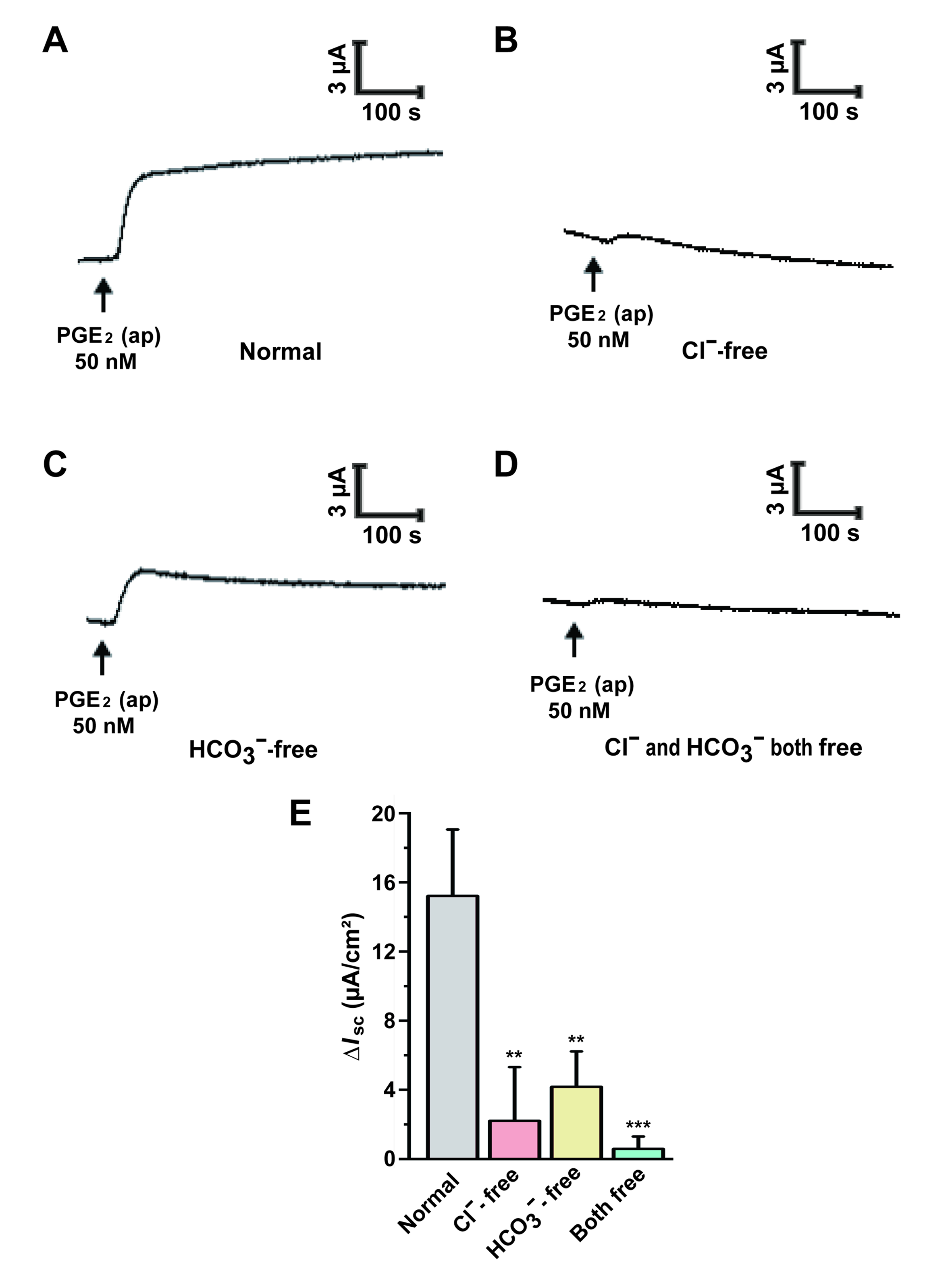

Supplement: S2 Fig — (A-D) Representative trace showing the ISC response induced by apical (ap) addition of PGE2 (50 nM) in normal K-H solution (A), Cl− -free K-H solution (B), HCO3−- free K-H solution (C), and Cl− and HCO3− both free K-H solution (D). (E) Statistical analysis showing the ISC response in rat vaginal epithelium induced by PGE2 (50 nM) in different K-H solutions. Symbols and bars indicate the mean ± S.D. (n = 3, ** P < 0.01, *** P < 0.001 versus the normal K-H solution group). (TIF) [file pntd.0009319.s002.tif]

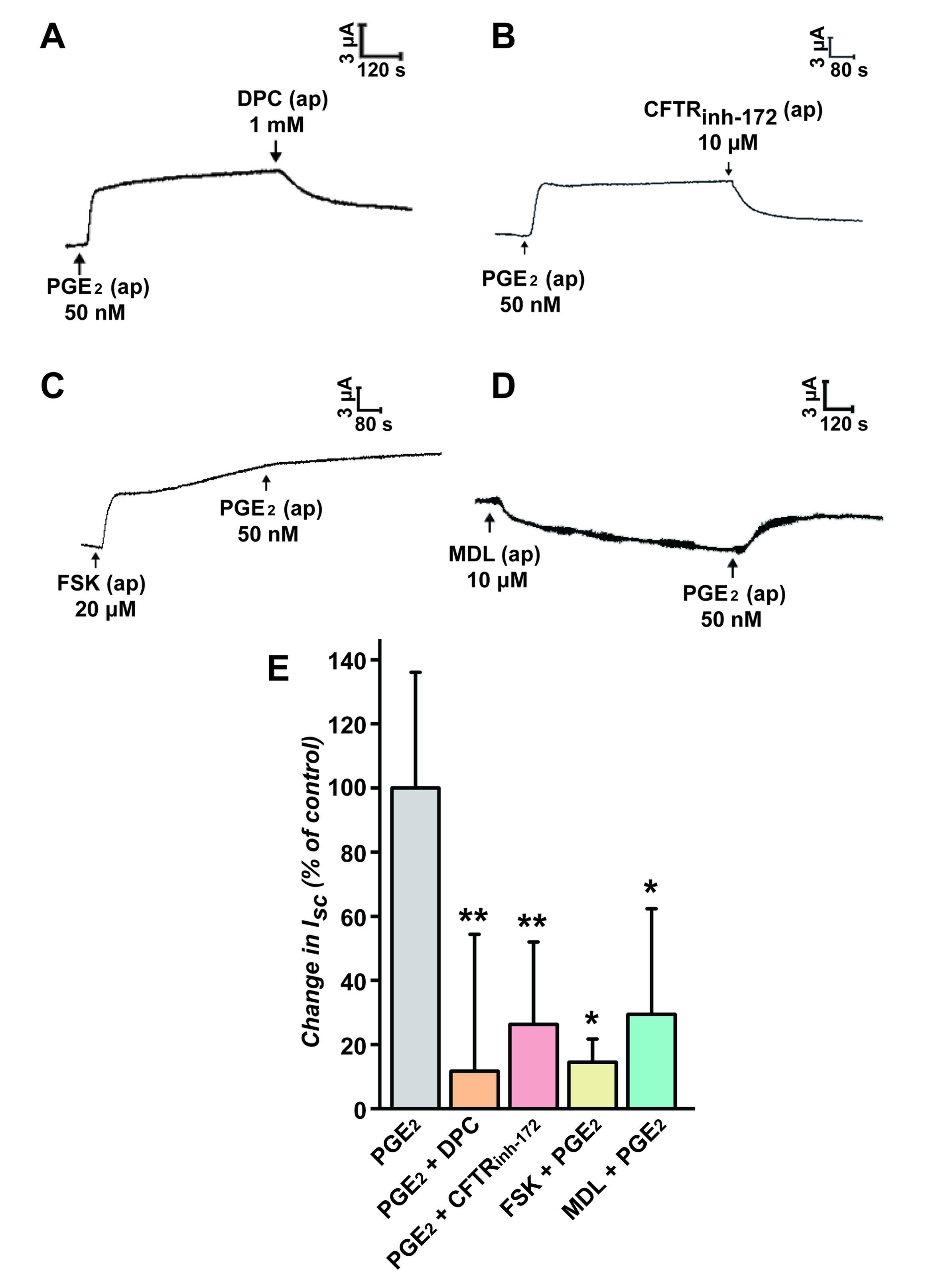

Supplement: S3 Fig — (A-D) Representative trace showing the short-circuit current (ISC) response induced by apical (ap) addition of PGE2 (50 nM) after treatment with the non-selective Cl− channels blocker DPC (1 mM, ap) (A), the selective CFTR blocker CFTRinh-172 (10 μM, ap) (B), the adenylate cyclase activator forskolin (FSK, 20 μM, ap) (C), or the adenylate cyclase inhibitor MDL-12330A (MDL, 10 μM, ap) (D). (E) Statistical analysis showing the effect of inhibitors on the Isc response induced by PGE2. Symbols and bars indicate the mean ± S.D. (n = 3–6, * P < 0.05, ** P < 0.01 versus the PGE2 group). (TIF) [file pntd.0009319.s003.tif]

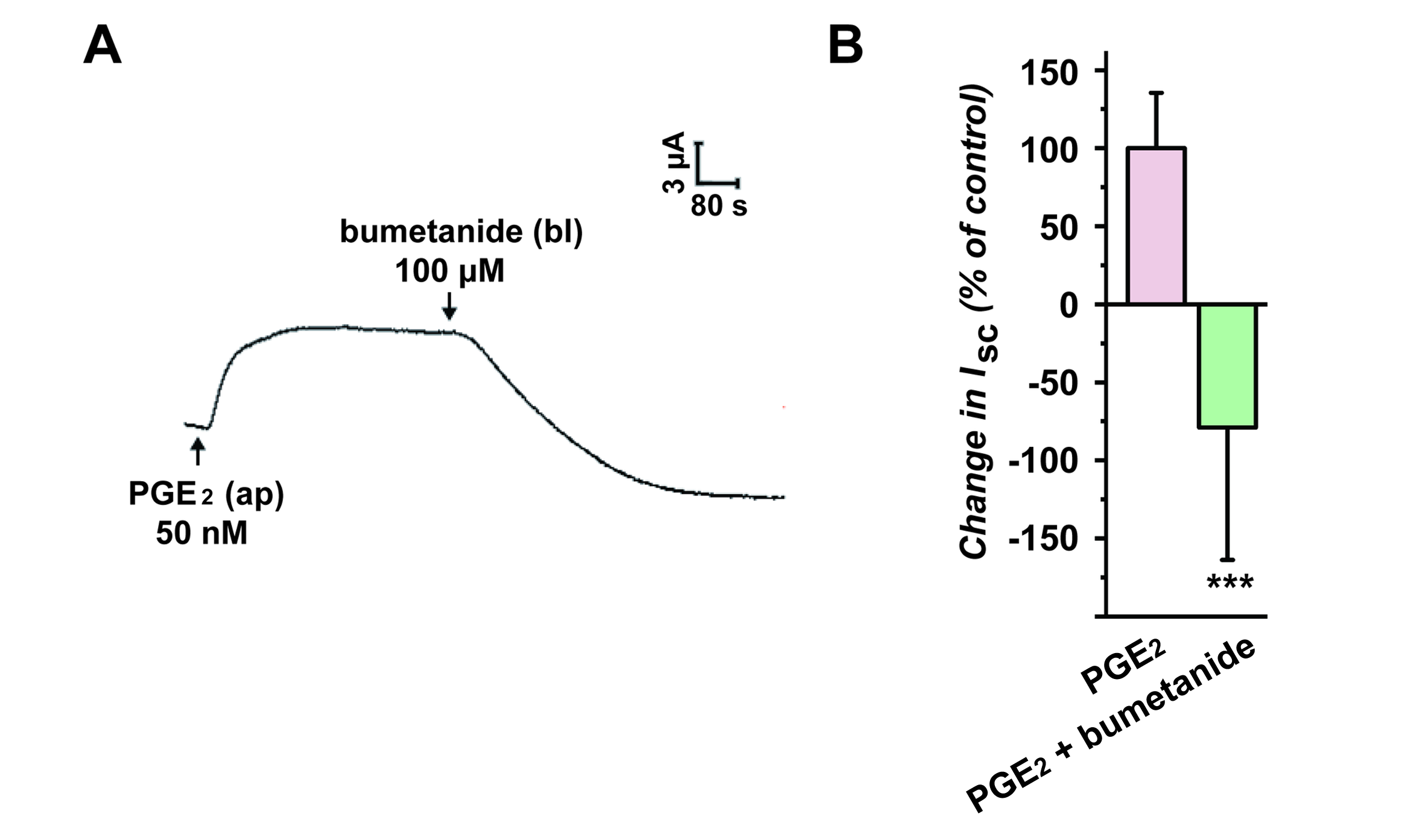

Supplement: S4 Fig — (A) Representative trace showing the ISC response induced by apical (ap) PGE2 (50 nM) in the presence of basolateral (bl) administration of bumetanide (100 μM), an inhibitor of the Na+-K+-2Cl− cotransporter, in rat vaginal epithelium. (B) Statistical analysis showing the effect of basolateral applied bumetanide on the ISC response induced by PGE2. Symbols and bars indicate the means ± S.D. (n = 4, *** p < 0.001 versus the PGE2 group). (TIF) [file pntd.0009319.s004.tif]

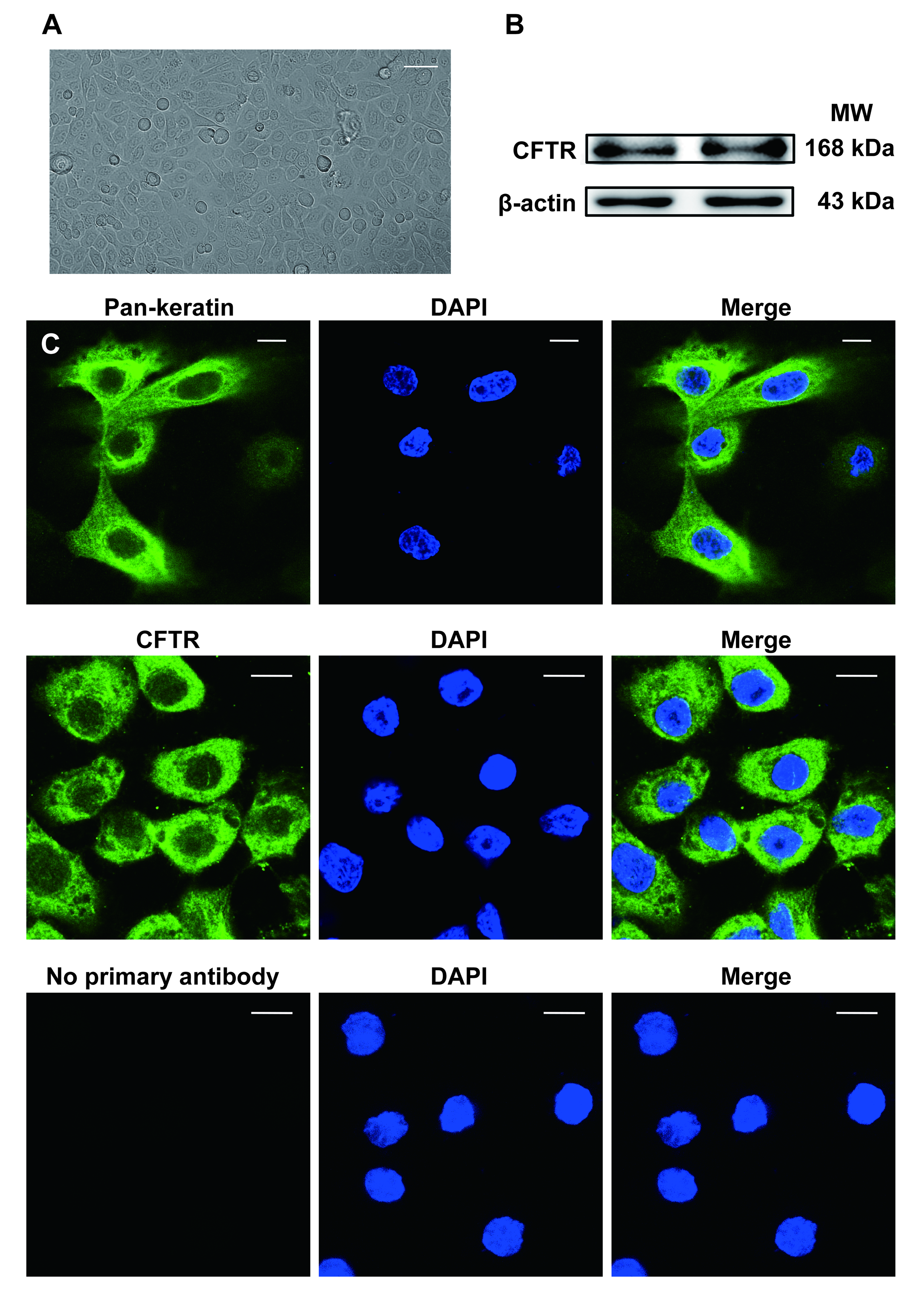

Supplement: S5 Fig — (A) Light microscope image of primary cultured rat vaginal epithelial cells (Day 5). Scale bar = 50 μm. (B) Representative blots showing the expression of cystic fibrosis transmembrane conductance regulator (CFTR) in primary cultured vaginal epithelial cells (Day 4). (C) Immunofluorescence images showing the expression of keratin, the marker of epithelial cells, and CFTR in primary cultured rat vaginal epithelial cells (Day 4), with the negative control. Scale bar = 10 μm. (TIF) [file pntd.0009319.s005.tif]
